# URANIUM IN SOIL AND GAMMA DOSE RATE AS PROXIES FOR THE INDOOR RADON RISK: SITUATION IN BELGIUM

**DOI:** 10.1093/rpd/ncx146

**Published:** 2017-09-23

**Authors:** F Tondeur, G Cinelli, B Dehandschutter

**Affiliations:** ncx1461 ISIB, Haute Ecole de Bruxelles-Brabant, rue Royale 150, B-1000 Bruxelles, Belgium; ncx1462 European Commission, DG Joint Research Centre, G10—Knowledge for Nuclear Safety, Security & Safeguards, via E. Fermi 2749, I-21027 Ispra, VA, Italy; ncx1463 Federal Agency for Nuclear Control, Ravensteinstraat 36, B-1000 Brussel, Belgium

## Abstract

Radon risk maps are usually based either on indoor radon data, or on measurements of soil gas radon and soil permeability. If these data are not available or not sufficient, it was suggested that other data could be used as an approximate substitute (a proxy) to the missing information, like the concentration of ^238^U or ^226^Ra in soils or the terrestrial gamma dose rate (TGDR). We examine here the correlation between airborne measurements of soil U and indoor radon, and between airborne U and TGDR, and their link with affected/unaffected areas. No clear correlation is found between airborne U and affected areas, as strongly affected areas are not characterised by a higher U level. Only the moderately affected area of Condroz can be connected to a higher U level, related to a few U anomalies. TGDR shows a rather good correlation with airborne U, but its relation with radon risk is less clear. Soil uranium and TGDR may help to screen out areas with very low U and very low TGDR, which have a low indoor radon risk, but they cannot be considered as good proxies for predicting radon-affected areas in Belgium.

## INTRODUCTION

It is known for more than 30 years that indoor radon is a major cause of exposure of the public to natural ionising radiation^(1, 2)^. The recent European Basic Safety Standards Directive^(3)^ presents several aspects concerning the protection against natural radioactivity in general and indoor radon in particular. In particular, ‘…Member States shall identify areas where the radon concentration (as an annual average) in a significant number of buildings is expected to exceed the relevant national reference level…’. The latter areas, traditionally called ‘radon-affected’ or ‘radon-prone’ and recently ‘radon-priority’ areas, can be identified using direct measurements of indoor radon or indirect quantities, such as soil gas radon concentration and soil permeability, which may be combined in the definition of a ‘geogenic radon potential’^(4)^. These data are not available everywhere with a good sampling density, and the proposal was made to relate the geogenic radon potential to other data which are expected to be correlated with it, like terrestrial gamma dose, uranium data and geological information^(5, 6)^. The methodology for connecting the geogenic radon potential with these data used as ‘proxies’ is not yet firmly established, and it is important at this stage to explore the expected correlations in order to optimise their use. Several Belgian databases allow a partial exploration:

– Indoor radon data, with a good density in the Walloon region (~1 data/km^2^) where all known radon-affected areas are located, less good for Flanders and Brussels (~0.1 data/km^2^) where no affected area was observed.
– A geological analysis of indoor radon data with the determination of the most appropriate geological units (GU) and which among them are radon-affected.
– Airborne surveys of K, Th and U reported as 100 data/km^2^. After calibration, these surveys can be used to calculate the terrestrial gamma dose rate (TGDR).

It is thus possible to study the correlations between indoor radon, radon-affected areas, geology, soil uranium and TGDR at a detailed scale. Unfortunately, a detailed database for soil radon and soil permeability in Belgium is only available for ~12% of the territory and will not be considered here.

## INDOOR RADON DATABASE AND GEOLOGICAL ANALYSIS

The Belgian indoor radon database was described in a previous work^(7)^. A total of 1119 long-term data collected in Brussels and in the Flemish region were added to the initial database limited to the Walloon region. The geological context was determined for each data and the correlation between indoor radon and geology was studied in detail^(8)^, a set of 35 GU appropriate for radon studies in Belgium being defined. With respect to this work, the present analysis includes a few changes:

–Unit DUL (Upper Devonian of Liège province) is integrated into DUC (Upper Devonian of Condroz) presenting a similar level of radon risk^(7)^.
–Unit TOU (Tournaisian) is separated into a Northern branch TNO and a Southern branch TCO^(7)^.
–Unit TER (Tertiary) is separated into SAN (Cenozoic sand) and CLA (Cenozoic clay), according to the dominant lithology of the considered formations.
–The quaternary cover, the limits of which are not displayed in the geological maps but which is only mentioned at the sampling points, was evaluated visually on the map for indoor radon data as well as for the kilometric grid used hereafter for airborne data, by looking at the nearest sampling points. This step includes a kind of ‘expert judgement’ which is not totally objective. The quaternary loess cover defines the GU QLO, whereas the sand cover is included in SAN.
–Quaternary valley alluvia are still discarded, for the reasons exposed in Ref.^(8)^.

A classification of the GUs into three categories of risk was given in Ref.^(7)^ based on the percentage of dwellings bypassing the reference level (400 Bq/m^3^ at that time): <1% unaffected, 1–10% moderately affected, >10% strongly affected. This classification is not very sensitive to the change of the reference level down to 300 Bq/m^3^, and will be kept unchanged here.

## AIRBORNE RADIOMETRIC SURVEY

An airborne radiometric survey was organised by the Belgian Geological Survey in 1994^(9)^. A short description was given recently^(10)^. The data are reported as counts/s on a 100 m × 100 m grid, and were not converted into soil concentrations of K, Th and U by the authors of the survey. The calibration of U data includes delicate problems that were discussed recently^(10)^.

The calibration of K and Th data is more straightforward. A detailed discussion will be given in a separate article^(11)^. A first step was to gather a database of local geochemical or radiometric measurements from different laboratories and measurement campaigns: FOREGS^(12)^, GEMAS^(13)^, University of Ghent^(14)^, SCK-IHE^(15)^. A total of 127 K data, and 145 Th data, was collected, all georeferenced. An harmonisation step, similar to the one described for U data^(10)^ was necessary because of the variety of measurement methods. Taking SCK-IHE as the reference, an harmonisation factor was applied to FOREGS, GEMAS and U.Ghent data. The airborne data were interpolated at the coordinates of harmonised data. Airborne data were slightly shifted for adjusting the result over the North sea, and a linear fit gave then the conversion factor of c/s into %(K) or ppm(Th).

## AIRBORNE U vs. INDOOR RADON, GEOLOGY AND AFFECTED AREAS

**Figure 1.**
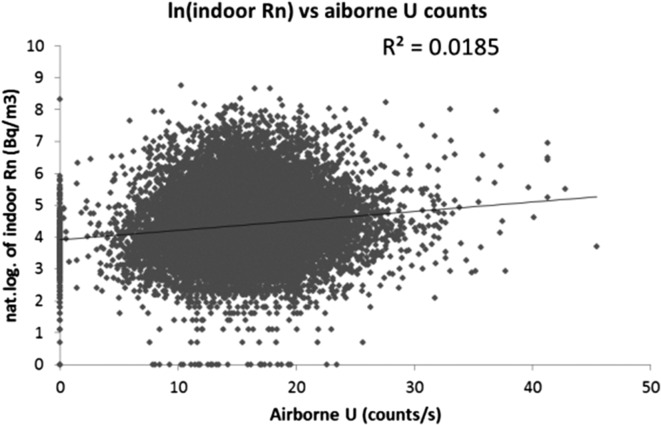
Natural logarithm of measured indoor radon concentration vs. airborne U at the same coordinates.

A first approach of the possible correlation between soil U and indoor radon, is to interpolate the raw airborne U data (in c/s) at the coordinates of indoor radon data. The plot of Rn vs. U (Figure 1) shows a nearly perfect absence of correlation (*R*^2^ = 0.02)! However, because of the lower sampling density in the Flemish region, the low U/low Rn category is underrepresented. Moreover, both datasets are extremely noisy. Smoothing was thus introduced in two different ways: (1) aggregating data according to the 37 GUs defined above, most of which being quite homogeneous for the indoor radon risk^(8)^, and (2) aggregating the data by 10 km × 10 km squares.

**Figure 2.**
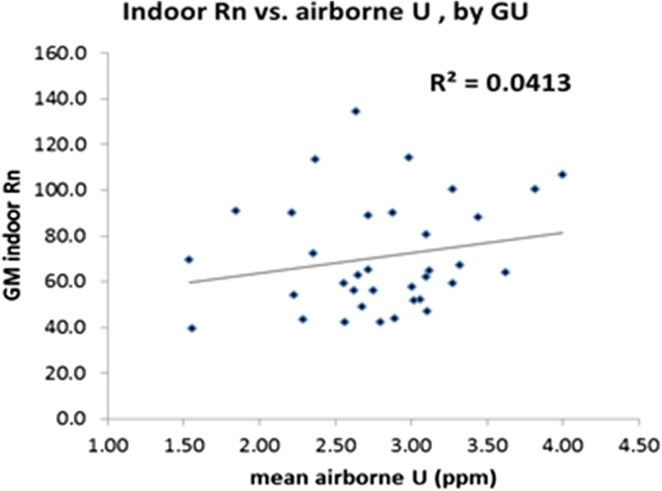
Geometrical mean indoor radon (Bq/m3) vs. mean airborne soil U (ppm) for geological units.

Table 1 gives mean values by GU, with a mention of the level of radon risk (unaffected, moderately or strongly affected). Aggregating data into GUs does not improve much the correlation between soil U and indoor Rn (Figure 2). Discarding the small GUs (<100 km^2^), with a hope of noise reduction, only slightly improves *R*^2^ to 0.08.

**Table 1.** Geometrical mean indoor Rn (Bq/m3), radon risk class (U = unaffected, M = moderately affected, S = strongly affected), mean airborne soil U (ppm), mean terrestrial gamma dose rate (nGy/h) for the different geological units.

Geological unit	Indoor Rn GM	Rn risk	Mean U	Mean TGDR
QLO	58	U	3.01	65
CLA	43	U	2.29	51
SAN	40	U	1.56	36
CRE	49	U	2.68	59
HSM	52	U	3.06	65
SME	114	S	2.98	73
CUB	90	S	2.22	54
ODY	100	S	3.28	57
CCM	72	S	2.35	64
CLM	42	U	2.56	63
OBR	56	U	2.75	64
SBR	42	U	2.80	70
DMB	44	U	2.89	73
DUB	47	U	3.11	73
TNO	63	M	2.65	57
VNO	65	M	2.72	60
OCO	52	U	3.02	74
SCO	89	U	2.72	75
DLC	54	U	2.23	60
DMC	62	M	3.10	70
DUC	67	M	3.32	78
TCO	64	M	3.62	74
VCO	100	M	3.82	72
TSE	59	U	3.27	73
CRO	69	U	1.54	49
PER	65	U	3.12	63
DUF	56	U	2.63	74
DMF	81	M	3.10	71
DLS	90	M	2.88	60
DLR	91	M	1.85	57
CST	113	S	2.37	50
OST	107	S	4.00	82
DLA	134	S	2.64	64
TRI	88	M	3.44	67
JUR	59	U	2.55	52

The main positive result is that the GU with the lowest mean airborne U, Cenozoic sand SAN, also has the lowest geometrical mean (GM) for indoor radon and is the main unaffected GU. No affected GU has a mean soil U less than 1.8 ppm, and no unaffected GU has a mean soil U higher than 3.27 ppm, but the most important strongly affected GU (Lower Devonian of Ardenne DLA) has a mean soil U of 2.64 ppm, in the middle of the range of soil U concentrations.

It is clear that in Belgium mean soil U would have little utility for predicting which GUs are affected (affected GUs above 3.27 ppm represent ~6% of the territory, ~25% of affected areas); it could nevertheless be useful for discarding some of the unaffected GUs (unaffected GUs under 1.8 ppm represent ~30% of the territory, ~40% of unaffected areas)

Among the affected GU groups defined in^(7)^, only Condroz (DMC + DUC + TCO + VCO) has a mean soil U > 3.27 ppm, partly connected to U anomalies in Dinantian (TCO + VCO). Interestingly, the main spot of high indoor radon in this area (at the centre of Figure 1 in Ref.^(7)^) also appears quite well in the airborne U map (Figure 9 in Ref.^(10)^). This nice illustration of a correlation between soil U and indoor radon however remains an exception!

Soil U is thus not the main parameter influencing indoor radon risk in Belgium. Other parameters to be considered are soil permeability, rock fracturation, but also rock weathering as discussed by Roth^(16)^, who showed in Eisleck, a region of Luxembourg in continuity with lower Devonian of Ardenne DLA, that the origin of the high radon risk is there the concentration of Uranium in weathering products, a result of rock history, not directly connected to its global uranium content.

The level of indoor radon risk can be explained, with the existing knowledge, for ~85% of Belgium (Table 2). More data, especially for permeability, should be collected for many small GUs that make the other 15%, especially DUF (upper Devonian of Fagne-Famenne) and JUR (Jurassic of Gaume).

**Table 2.** Gross classification of areas according to the radon risk with the main explanation.

Area	Surface (%)	Explanation
Unaffected Cenozoic sand	~30	Low uranium
Other unaffected Cenozoic	~33	Low permeability K
Strongly affected Ardenne	~17	Medium/high K, fractures, weathering
Affected Condroz exc.DMC	~5	Medium/high uranium
Other affected	~2	?, not high U
Other unaffected	~13	?, not low U

**Figure 3.**
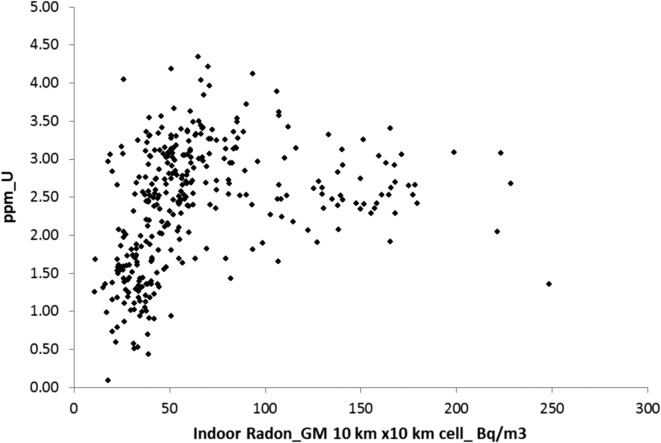
Mean of airborne uranium concentration vs. geometric mean of indoor radon concentration data over 10 km × 10 km cells.

Figure 3 reports the correlation between the geometrical mean of indoor radon data and the mean of U airborne data over 10 km × 10 km cells. The grid is the one defined by the JRC for the European Indoor Radon Map^(17)^. The statistics of indoor radon data on this grid has been updated by FANC in July 2016.

Figure 3 shows a higher variability of U (between the minimum, 0.08 ppm, and maximum, 4.34 ppm) for low indoor radon concentration (<75 Bq/m^3^) than for high indoor radon concentration (>75 Bq/m^3^) where U ranges between 1.5 and 3.5 ppm. This suggests that other parameters in addition to U concentration in soil must be invoked for understanding the indoor radon value. However some useful information from this plot could be extracted: high indoor Rn concentrations could only be reached if a certain amount of U is present. Below 1.5 ppm of U the indoor radon GM should be less than 50 Bq/m^3^ with very few exceptions.

## TGDR vs. AIRBORNE U, INDOOR RADON, GEOLOGY AND AFFECTED AREAS

The TGDR can be calculated from the soil concentrations of K, Th and U determined from airborne data^(2)^.

Because there is a significant correlation between U on the one hand, K and Th on the other, TGDR is rather well correlated with soil U (correlation coefficient = 0.84). To some extent, TGDR could thus be considered as a proxy for soil U, and we can expect some similarity when both are put against indoor radon, geology and affected areas. This is the case with, of course, some additional blurring of the information.

Table 1 shows that SAN, the main unaffected GU with low U/low Rn, also has the lowest TGDR. No affected GU is found under 50 nGy/h, but the second lowest TGDR is observed for CST, a strongly affected GU! Similarly, the two highest TGDR are for OST, strongly affected, and DUC, moderately affected, but the next three GUs by TGDR (SCO, OCO, DUF) are unaffected.

The plot of the mean TGDR vs. indoor radon GM for 10 km × 10 km cells is very similar to Figure 3. With few exceptions, cells with TGDR < 40 nGy/h have low Rn.

## CONCLUSION

The concentration of U in soil, and the TGDR, are not clearly correlated in Belgium with indoor radon levels and are not good proxies for predicting radon-affected areas. Their main utility in this context would be to screen out areas with low U (<1.5 ppm) or low TGDR (<40 nGy/h) which most probably are not radon-affected.
